# A New Conjugation Method Used for the Development of an Immunoassay for the Detection of Amanitin, a Deadly Mushroom Toxin

**DOI:** 10.3390/toxins10070265

**Published:** 2018-06-28

**Authors:** Candace S. Bever, Bogdan Barnych, Robert Hnasko, Luisa W. Cheng, Larry H. Stanker

**Affiliations:** 1 Foodborne Toxin Detection and Prevention Unit, Western Regional Research Center, Agricultural Research Service, United States Department of Agriculture, 800 Buchanan Street, Albany, CA 94710, USA; candace.bever@ars.usda.gov (C.S.B.); luisa.cheng@ars.usda.gov (L.W.C.); 2 Department of Entomology and Nematology, UC Davis Comprehensive Cancer Center, University of California-Davis, Davis, CA 95616, USA; bbarnych@ucdavis.edu; 3 Produce Safety and Microbiology Unit, Western Regional Research Center, Agricultural Research Service, United States Department of Agriculture, 800 Buchanan Street, Albany, CA 94710, USA; robert.hnasko@ars.usda.gov

**Keywords:** amatoxin, amanitin, death cap mushroom, *Amanita phalloides*, ELISA, immunoassay

## Abstract

One of the deadliest mushrooms is the death cap mushroom, *Amanita phalloides*. The most toxic constituent is α-amanitin, a bicyclic octapeptide, which damages the liver and kidneys. To develop a new tool for detecting this toxin, polyclonal antibodies were generated and characterized. Both α- and β-amanitin were coupled to carrier proteins through four different linking chemistries, one of which has never before been described. These conjugates were evaluated for their effectiveness in generating antibodies specific for the free toxin, as well as their utility in formatting heterogeneous assays with high sensitivity. Ultimately, these efforts yielded a newly described conjugation procedure utilizing periodate oxidation followed by reductive amination that successfully resulted in generating sensitive immunoassays (limit of detection (LOD), ~1.0 µg/L). The assays were characterized for their selectivity and were found to equally detect α-, β-, and γ-amanitin, and not cross-react with other toxins tested. Toxin detection in mushrooms was possible using a simple sample preparation method. This enzyme-linked immunosorbent assay (ELISA) is a simple and fast test, and readily detects amatoxins extracted from *A. phalloides*.

## 1. Introduction

**Figure 1:**
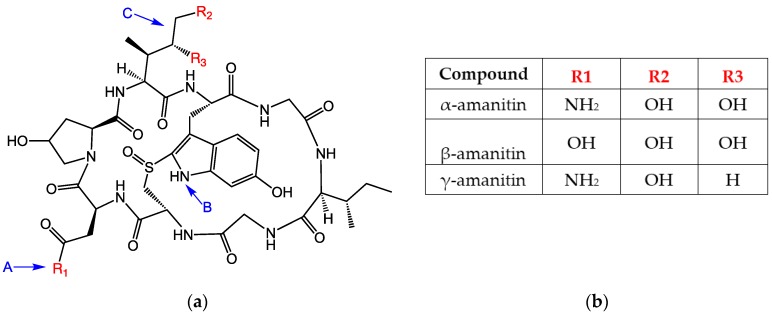
Chemical structures of the amatoxin variants examined in this paper. (**a**): The putative binding locations for each hapten is indicated by blue arrows and letters. (**b**): R group substituents for the three amatoxins listed.

The most dangerous and poisonous mushroom is the death cap, *Amanita phalloides*. *A. phalloides* are widely distributed around the world, predominantly thought to be an invasive species originating from Europe and now it has been identified on every continent except Antarctica [1]. *Amanita* mushrooms contain several toxins, however the bicyclic octapeptides known as amatoxins, and in particular α-amanitin (α-AMA) is considered the principle toxin (Figure 1). Amatoxins are highly stable, impervious to heat, cold, or acid treatments. Amatoxins are potent inhibitors of the transcription process via inhibition of RNA polymerase II. *A. phalloides* contains variable amounts of toxins based on growth phase, location, environmental conditions, and tissue type [2]. In one report, a single, fresh mushroom, weighing 14.8 g was found to contain 5.36 mg of total (α-AMA, β-AMA, and γ-AMA) amatoxins [3]. The LD_50_ for mice is 0.1 mg/kg [4] and α-AMA is severely toxic to humans at 0.3 mg/kg [5].

Current methods for quantifying amatoxins include high performance liquid chromatography (HPLC) with detection by either ultraviolet (UV) or mass spectrometry (MS) [3,6], thin-layer chromatography (TLC) [7], capillary zone electrophoresis [8], or immunoassay [9,10,11]. The most sensitive HPLC method with a UV detector provides a detection limit of 10 µg/L of extraction buffer (corresponding to 0.5 ng/g mushroom tissue) [3]. A plate-based immunochemical method reports a limit of detection (LOD) of 1.9 µg/L [12] and a polyclonal sheep immunoassay reported a detection limit of 0.08 µg/L [9].

Although the toxin itself is not immunogenic because of its low molecular weight, it can be made immunogenic by linking to a larger carrier molecule, usually a protein such as bovine serum albumin (BSA) or keyhole limpet hemocyanin (KLH). Such conjugates are referred to as antigens or immunogens, while the small molecules used for conjugation are called haptens. Thus, immunoassays for α-AMA are achievable because the toxin can be covalently attached to a larger protein resulting in inducing a hapten-specific immune response. Amatoxins present various functional groups that have been used for covalent conjugation to a larger carrier protein. The functional group most often utilized is the carboxyl group on β-AMA (position R1 in Figure 1, arrow A). The earliest studies attempting to generate antibodies to amatoxins used β-AMA conjugated to protein carriers using ester or amide linkages [9,10,13,14] (Figure 1, arrow A). Some of these earlier studies found the resulting material to be toxic to the animals immunized. Another study used β-AMA as the starting material and created a cyanuric chloride (CC) bridge, presumably with the indole [9] (Figure 1, arrow B). Phenol function in β-AMA was also exploited for conjugation either by direct reaction with succinic anhydride or by its preliminary alkylation followed by deprotection of the introduced amine and reaction with succinic anhydride (MA) [11,15] (Figure 1, arrow A). Periodate oxidation (PERI) of the terminal diol (from the dihydroxylated isoleucine) (Figure 1, arrow C) in α-AMA has been used to attenuate toxicity [16]. To our knowledge, no studies have explored using periodate oxidation to couple α-AMA with a carrier protein for the purpose of generating antibodies. Cleaving at the diol group generates a reactive aldehyde, which can be subjected to reductive amination to form a stable amine linkage with an available amine on the carrier protein. 

We hypothesize that we can utilize periodate oxidation as a linking chemistry to generate antibodies to amatoxins. In this study, we test this hypothesis by generating the immunogens, immunizing rabbits, and characterizing the induced antibodies for reactivity towards free amatoxins. In addition, building on previous knowledge that some homologous haptens cannot be competed against with the free toxin [9], we constructed a panel of heterologous haptens using a variety of linking chemistries (Table 1) and utilized them in the development of competition enzyme-linked immunosorbent assays (cELISAs).

## 2. Results

### 2.1. Antibody Activity

Sera from rabbits obtained at week 13 post-immunization were screened by ELISA to determine antibody titer. Evaluation of the immune response against each conjugate is shown in Table 2a. All of the animals produced measurable antibody responses to their homologous coating antigens (AMA-BSA). The highest serum titers were obtained from rabbits immunized with haptens using the CC and LB linkage chemistries. Relatively weaker titers were obtained from the MA and PERI linkage chemistries. Sera from the CC-AMA-KLH immunized rabbits did not bind to the three other coating antigens. Serum from only one of the animals immunized with MA-AMA-KLH bound both the MA and CC conjugated antigens. In contrast the animals immunized with either the PERI or LB conjugates produced sera that bound all of the antigens regardless of the linkage chemistries.

Each serum-antigen pair that produced a signal by ELISA was screened for detection of free α-AMA by cELISA (Table 2b). The sera from the CC-AMA-KLH and MA-AMA-KLH immunized animals did not detect α-AMA at any concentration tested when the respective AMA-BSA conjugate with the homologous linkage chemistry was used as the coating antigen. Similar characteristics were observed for the one MA-AMA-KLH immunized rabbit (ID #65) whose serum produced a signal with the CC-AMA-BSA coating antigen, but still resulted in no α-AMA detection.

Conversely, antibodies derived from the PERI-AMA-KLH immunized animals could detect α-AMA regardless of the linkage chemistry used to produce the coating antigen (Table 2b). The inhibitory concentration at half of the maximum signal (IC_50_) ranged from 0.06 to 0.42 µg/L with minimal differences between the two rabbits. The one exception was the assay generated from one animal’s serum and using the LB-AMA-BSA coating antigen, which resulted in an IC_50_ of 2.98 µg/L.

Antibodies derived from the LB-AMA-KLH immunized animals could detect free α-AMA by cELISA, but the linkage chemistry used to produce the coating antigen exhibits a profound effect on assay sensitivity (Table 2b). For instance, free α-AMA was not detected when the homologous LB-AMA-BSA coating antigen was used in the cELISA. When the heterologous CC-AMA-BSA coating antigen was used, individual animals responded vastly different, such that one produced an assay with an IC_50_ of 0.48 µg/L, while the other produced an assay with an IC_50_ value 200-fold higher. The most sensitive assays (IC_50_ = 0.07 and 0.47 µg/L), from the antibodies derived from the LB-AMA-KLH immunized animals, were obtained when the PERI-AMA-BSA coating antigen was used, while the MA-AMA-BSA coating antigen assays resulted in IC_50_ values ranging from 5.97 to 8.65 µg/L.

**Figure 2:**
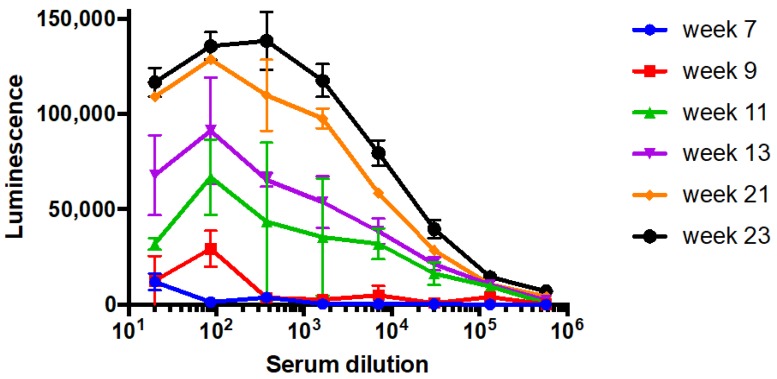
A representative ELISA serum titration sampled at six different time points. Rabbit (#58) serum after immunizations with LB-AMA-KLH using PERI-AMA-BSA as the coating antigen coated at 1 µg/mL.

We further investigated the two most sensitive sera/antigen combinations: PERI-AMA-KLH derived serum/CC-AMA-BSA antigen; and LB-AMA-KLH derived serum/PERI-AMA-BSA antigen. Both sets of rabbits (PERI-AMA-KLH and LB-AMA-KLH) were immunized with two additional booster injections and then larger volume terminal bleeds at week 23 were collected and used in the remainder of the studies. The serum titers continued to elevate in these animals following each subsequent boost and bleed. Figure 2 shows a representative ELISA titration of serum obtained from rabbit #58, immunized with the LB-AMA-KLH immunogen, screened by ELISA against the PERI-AMA-BSA coating antigen. 

### 2.2. Analyte Cross-Reactivity and Sensitivity

**Figure 3:**
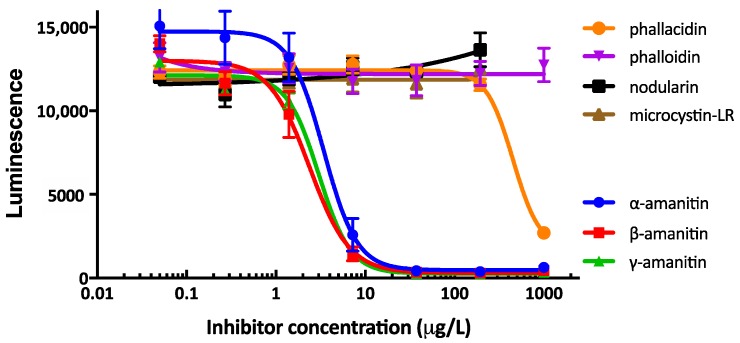
A representative competitive inhibition ELISA of serum antibodies from rabbit #58 binding to coating antigen PERI-AMA-BSA. The inhibitors were α-amanitin, β-amanitin, γ-amanitin, microcystin-LR, nodularin, phalloidin and phallacidin. Errors bars are shown from triplicate analyses.

The cross reactivity observed in both cELISAs (PERI-AMA-KLH derived sera/CC-AMA-BSA antigen and LB-AMA-KLH derived sera/PERI-AMA-BSA antigen) to related mushroom toxins and other cyclic peptide toxins was determined. Both cELISAs gave similar results. The results from the cELISA using rabbit serum #58 LB-AMA-KLH and coating antigen PERI-AMA-BSA are shown in Figure 3. Of the toxins tested, α-AMA, β-AMA and γ-AMA competitively inhibited without varying significantly (IC_50_ = 3.0, 2.0, and 2.6 µg/L, respectively). The overall range of detection (defined as IC_20_–IC_80_) is very sharp and covers from 0.3 to 7 µg/L. Phallacidin was detectable at a 1000-fold higher concentration than the amatoxins, while phalloidin, microcystin-LR, and nodularin, did not cross-react.

### 2.3. Assay Matrix Effects

**Figure 4:**
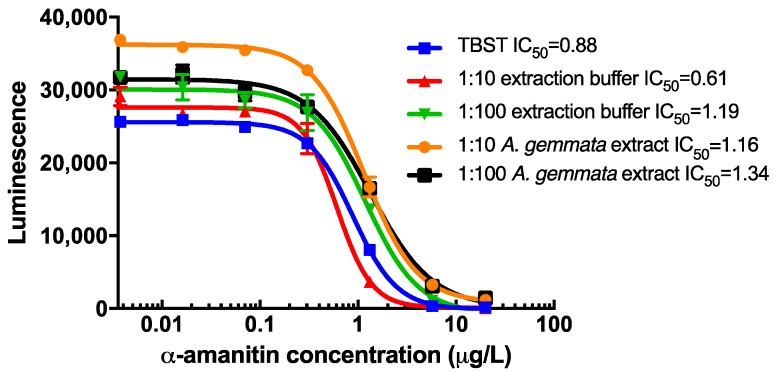
Matrix effect of extraction buffer and extracts from *A. gemmata* on the assay sensitivity. IC_50_ values (µg/L) are indicated in the legend. TBST = tris buffered saline with tween.

In order to evaluate matrix effects, assay performance using tris buffered saline with tween (TBST) versus extraction buffer (methanol:water:0.01 M HCl, 5:4:1, *v:v:v*) diluted 1:10 and 1:100 spiked with α-AMA were compared (Figure 4). Similar IC_50_ values were observed in these experiments. Next, a mushroom extract from *A. gemmata*, a species known to not contain amatoxins, was spiked with α-AMA and then evaluated by cELISA (Figure 4). The extraction buffer was slightly acidic, pH of 6.0 when diluted 1:1 with antibody serum in the final test. Limited matrix effects were observed in the maximum signal asymptote using both the extraction buffer and the *A. gemmata* extract at 1:10 and less so at 1:100 (Figure 4). Despite these small changes in absolute signal, the resulting IC_50_ values ranged from 0.61 to 1.34 µg/L. Additionally, parallelism between the slope of each curve is well maintained. While the assay exhibits approximately 30% variability between all of these conditions at the maximum signal, detection for amatoxin at or above 1 µg/L is reliable due to the steepness of each curve.

### 2.4. cELISA Analysis of Mushroom Extracts

**Figure 5:**
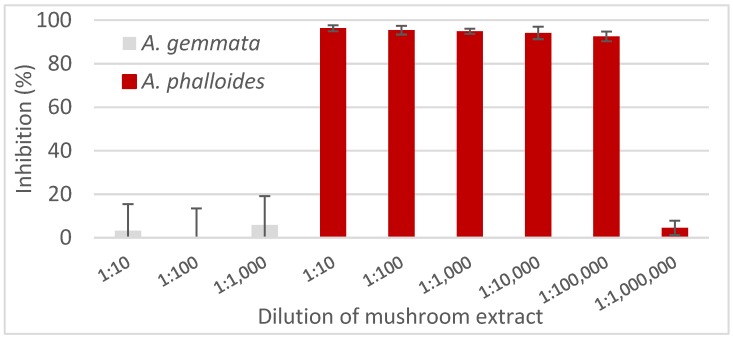
Analysis of mushroom extracts for amatoxins. Two representative mushroom extracts, *A. phalloides* and *A. gemmata*, were diluted as indicated and analyzed by cELISA.

We did not attempt to quantify the total amount of toxins in the entire mushroom, but instead tested if the cELISA could detect amatoxins in a sample from the caps of *A. phalloides* and *A. gemmata* used as a negative control. Homogenized tissue samples were extracted and then diluted in a 1:10 series to determine when the toxin concentration in *A. phalloides* would fall within the range of our standard curve. As shown in Figure 5 the only mushroom extract exhibiting inhibition in the cELISA was the extract from the *A. phalloides* sample and this was observed in samples diluted from 1:10 to 1:100,000.

## 3. Discussion

A new conjugation strategy, utilizing periodate oxidation (PERI) followed by reductive amination, has been successfully employed for the development of an immunoassay for amatoxin detection. While evaluating the new method, a panel of immunogens and coating antigens were generated using previously described conjugation methods (MA and CC) and a commercial hapten (LB). Antibodies were raised to all four different hapten conjugates, but only the PERI-AMA-KLH serum antibodies resulted in assays where either homologous or heterologous coating antigens can be used (Table 2). Alternatively, LB-AMA-KLH serum antibodies generated assays that only detected free toxin when a heterologous coating antigen hapten linkage chemistry was used (Table 2). This is similar to previous findings [9] wherein the MA linkage chemistry was used to generate the immunogen.

Antibodies described in this paper detect α-AMA, β-AMA, and γ-AMA with similar sensitivity and do not recognize other related toxins tested. Our results are thus similar to those reported earlier [10], but differ from mouse monoclonal antibodies that recognize α-AMA (100%), β-AMA (67%), and γ-AMA (39%) [11], sheep polyclonal antibodies that recognize β-AMA (100%) and α-AMA (22%) [9], as well as the commercially available amanitin test whose product insert states it is optimized to detect α-AMA (100%) and γ-AMA (90%) and has no cross-reactivity to β-AMA (0.1%) [17]. Because the location of the only difference in the structure of α-AMA and β-AMA (Figure 1) is not altered using this PERI conjugation method, it represents perhaps the best approach to produce monoclonal antibodies that distinguish α-AMA from β-AMA.

The minimal changes to the standard curve due to matrix effects on the performance of the amatoxin immunoassay observed in this study were also found in other studies evaluating matrices derived from mushroom extracts [11] and biospecimens [9]. It is unclear if the other studies observed changes to the absorbance maximum for each matrix type, as we show in Figure 4, since they plotted their data as a percentage of the maximum signal. Nonetheless, the parallelism of the curves we observed suggests that while there is some importance in selecting an accurate matrix as the standard curve, the range of quantitation would remain nearly the same. Because of the steepness of the curves obtained in this study, a qualitative assay with a cut-off of approximately 1 µg/L is probably the most accurate use of this assay. It is interesting to note that the IC_50_ values obtained during the screening process (Table 2) are lower than those determined in the final assay (Figure 4). This may be due to changes in antibody titer and affinity between the bleeds from week 13, when the screening was done, and week 23, when the larger volume of serum was collected for the final immunoassay (Figure 2).

The test described here accurately identifies the presence of amatoxins in the extract obtained from an *A. phalloides* mushroom and gave negative results for extracts from *A. gemmata* a species known not to contain toxin. Detection in *A. phalloides* extracts diluted 1:100,000 was expected, given that a single dry mushroom cap and gills may contain 6–7 mg of amatoxins/g of dried mushroom tissue [18]. In this study, extraction of 200 mg of dried mushroom in 2 mL of extraction buffer, followed by a 100,000-fold dilution should contain approximately 6 µg/L of toxin, well above the detection limit of this cELISA.

## 4. Conclusions

Four different conjugation chemistries were used to generate antibodies to amatoxins. Only two of the approaches, the PERI and the LB conjugates, resulted in antibodies that bound free amatoxins and were useful for developing a cELISA for these toxins. The antibodies generated following immunization with the PERI- and LB-conjugates behaved similarly with respect to sensitivity and selectivity. The resulting cELISA was able to detect α-AMA, β-AMA, and γ-AMA with similar sensitivity. To our knowledge this study represents the first report of a previously undescribed immunogen involving periodate oxidation of the terminal diol structure on α-AMA. It is reasonably anticipated that this method would also work for β-AMA, because it too contains the terminal diol (from the dihydroxylated isoleucine). In contrast, γ-AMA does not contain a terminal diol.

The cELISA described was able to detect amatoxins at levels as low as 1 µg/L. Furthermore the assay readily detected toxin in extracts of *A. phalloides* but not in extracts from non-toxin containing *A. gemmata* mushrooms. The simplicity of the extraction method and immunoassay suggest that this would be a candidate for a field portable test. Such a test becomes useful as *A. phalloides* continues to invade new geographic regions and since de novo chemical synthesis of the toxin has recently been described [19]. Studies underway include generation of monoclonal antibodies to these toxins and further application of the cELISA to detect amatoxins in mushroom populations.

## 5. Materials and Methods

### 5.1. Materials

The toxins, α-AMA (≥90%), β-AMA (≥90%), γ-AMA (≥90%), microcystin-LR (≥95%), nodularin (≥95%), and phalloidin (>90%), were purchased from Enzo Life Sciences (Farmingdale, NY, USA). Phallacidin (≥85%), sodium periodate (NaIO_4_, >99.8%), sodium borohydride (NaBH_4_, 99%), dimethyl sulfoxide (DMSO), and bovine serum albumin (BSA) were purchased from Sigma-Aldrich (St. Louis, MO, USA). Methanol, Tween-20, keyhole limpet hemocyanin (KLH), and SuperSignal West Pico PLUS chemiluminescent substrate were purchased from Fisher Scientific (Waltham, MA, USA). Secondary antibody goat-anti-rabbit-horseradish peroxidase conjugate was purchased from Abcam (Cambridge, MA, USA). The hapten OSu-Glu-vc-PAB-C6-amanitin (LB) was purchased from Levena Biopharma (San Diego, CA, USA).

### 5.2. Buffers

Phosphate buffered saline (PBS) was 10 mM phosphate and was used at pH 7.4, unless otherwise noted. Carbonate buffer (CB) was 0.05 M carbonate-bicarbonate and was used at pH 9.6, unless otherwise noted. Wash buffer (TBST) was 20 mM tris-buffered saline, pH 7.4, containing 0.05% Tween-20. Blocking solution was 3% non-fat dry milk in TBST.

### 5.3. Specimen Colleciton

Mushroom specimens were collected from the Point Reyes National Seashore in accordance with the National Park Service-issued Scientific Research and Collecting Permit (#PORE-2017-SCI-0054). Two varieties (*A. phalloides* and *A. gemmata*) were collected and identified by physical morphological characteristics (white free gills, bulbous volva, cap color, etc.). All mushroom identifications were confirmed by a trained mycologist and cross-referenced in *Mushrooms of the Redwood Coast* [20]. A white spore print was generated from *A. phalloides* to further confirm its identification. The mushroom cap was laid gill-side down on black paper for 24 h at room temperature. All remaining mushrooms were dehydrated in an oven at 65 °C for 48 h. The dehydrated caps (pileus and gills) were stored at room temperature in a desiccator until used.

### 5.4. Hapten-Protein Conjugation

Conjugates of amatoxins with BSA and KLH were prepared to serve as coating antigens and immunogens, respectively. Four different conjugation chemistries were performed and are summarized in Table 1. β-AMA was conjugated using the mixed anhydride (MA) and cyanuric chloride (CC) bridging methods as described [9]. For both the MA and CC methods, 1 mg of activated β-AMA was reacted with 10 mg of BSA and 0.5 mg of activated β-AMA was reacted with 7 mg of KLH. The commercially available α-AMA hapten termed ‘LB’ (OSu-Glu-vc-PAB-C6-amanitin) was conjugated through its terminal N-hydroxysuccinimide ester as follows. The LB hapten was dissolved in DMSO to a final concentration of 3 μM and 50 μL was added to 6.6 mg BSA (10 mg/mL in PBS, pH 8.2) and 100 μL was added to 10 mg KLH (10 mg/mL in PBS, pH 8.2). 

The fourth conjugation method was the periodate oxidation (PERI) of the terminal diol of α-AMA following the general method described in [21]. Briefly, α-AMA (1 mg) was dissolved in distilled water (500 μL). NaIO_4_ (218 μL, 40 mM in water) was added to the α-AMA solution and stirred for 20 min in the dark at room temperature. A color change from faint yellow to a deep golden yellow was observed as the reaction proceeded. The activated α-AMA solution (350 μL) was then added to 3 mg BSA (10 mg/mL in CB, pH 9.0) and to 2 mg KLH (10 mg/mL in CB, pH 9.0) and stirred for 2 h in the dark at room temperature. NaBH_4_ (25 μL, 0.1 M, in 10 mM NaOH) was added and the resulting mixture was stirred for 2 h in the dark at room temperature. 

Individual hapten-protein conjugates were dialyzed against 50 mL of PBS using a 10 K MWCO, Slide-A-Lyzer MINI Dialysis Device (Fisher) with 3 exchanges in a 24-h period. Protein concentration for all hapten conjugates was determined on a NanoDrop Lite Spectrophotometer (Fisher). The conjugates were stored at −20 °C.

### 5.5. Immunization and Antibody Production

All animal studies were conducted in accordance with protocol (#1) approved by the Pacific Immunology Corp. (Ramona, CA, USA) institutional animal care and use committee. New Zealand White Rabbits (two for each immunogen) were immunized with 200 μg of AMA–KLH mixed 1:1 with Complete Freund’s Adjuvant. Booster injections were administered approximately every three weeks at a dose of 100 μg of AMA–KLH mixed 1:1 with Incomplete Freund’s Adjuvant. A total of four booster injections were administered. Production bleeds (#1–4) were collected one and three weeks after the third and fourth booster immunizations. The two sets of animals with the most promising antibody response (as determined below), were administered two additional boosts and bleeds (#5–6) were collected two weeks after the second booster immunization and at exsanguination.

### 5.6. Antibody Activity and Assay Characterization

Each anti-serum was screened for antibody activity by indirect ELISA against each of the four AMA-BSA coating antigens. Wells of black high-binding microtiter plates were coated with antigen at 1 µg/mL (100 µL/well) in CB for 1 h at 37 °C with gentle rocking. Wells were blocked with 3% non-fat dry milk in TBST. Serum (100 µL/well) in triplicate was serially diluted in TBST and incubated for 1 h at 37 °C with gentle rocking. Plates were then washed with three rinses of TBST. Secondary antibody was added at 1:50,000 (100 µL/well) in TBST. Again the plates were washed with three rinses of TBST and developed using a chemiluminescent substrate. The plates were incubated for 3 min with gentle rocking and then luminescence was read on a Victor plate reader (Perkin Elmer, Waltham, MA, USA).

Each serum that reacted with a coating antigen was used in a cELISA in order to identify which pairs of serum and coating antigen result in detection of the free α-AMA toxin. Plates were prepared as described above, coated at 1 µg/mL. Dilutions (50 µL/well) of α-AMA in triplicate were prepared in TBST starting at 1000 µg/L and diluted fivefold, with control wells only loaded with TBST and no inhibitor. Sera (50 µL/well) in TBST at a concentration of two times the EC_50_ was loaded. Plates were incubated for 1 h at 37 °C with gentle rocking. Plates were then washed, incubated with secondary antibody, washed again, and developed as described above.

Assay characterization was completed for the two pairs of antibody and coating antigen with the most sensitive α-AMA detection in these initial experiments. First, titers of all production bleeds (#1–6) were obtained by ELISA. The sera from the week 23 exsanguination bleed (#6) was used for the remaining experiments. The sera from the LB-AMA-KLH immunized rabbits were screened against PERI-AMA-BSA coating antigen and the sera from the PERI-AMA-KLH immunized rabbits were screened against CC-AMA-BSA coating antigen for both the titer determination and the remaining experiments. Next, a checkerboard titration was completed to determine the minimal concentrations of serum and coating antigen that result in a suitable signal above background. With these determined conditions, cELISAs were completed using a panel of inhibitors, including α-AMA, β-AMA, γ-AMA, phalloidin, phallacidin, microcystin-LR, and nodularin. Each inhibitor was initially dissolved in water and then diluted into TBST. Each inhibitor (50 µL/well) was tested in triplicate starting at 1000 µg/L and diluted fivefold in TBST, with control wells only loaded with TBST and no inhibitor. Again, antibody serum (50 µL/well) was added at two times the EC_50_ value as determined by checkerboard ELISA and added equally to all wells. Plates were incubated for 1 h at 37 °C with gentle rocking. Plates were washed, incubated with secondary antibody, washed again, and developed as described above. 

### 5.7. Mushroom Extraction

Dried mushroom samples (4 *A. phalloides* and 2 *A. gemmata*) were extracted with slight modifications to previously published methods [3,6]. Briefly, dried mushroom cap tissue was ground to a powder with a mortar and pestle. Approximately 200 mg of this powder was then suspended with 2 mL of extraction buffer (methanol:water:0.01 N HCl, 5:4:1, *v:v:v*), shaken for 1 h at room temperature, and then centrifuged at 1000× *g* for 10 min. Aliquots of the supernatant were diluted in TBST and analyzed by cELISA.

To determine if the mushroom extracts exhibited any matrix effects, cELISA tests were completed following extraction of *A. gemmata* (a non-toxin containing member of the genus). The extract was then diluted at 10- and 100-fold. Aliquots of the extracts, in triplicate, were then spiked with α-AMA at varying concentrations ranging from 20 to 0.02 µg/L including a non-spiked blank. Other assay matrices tested were TBST as well as a 10- and 100-fold dilution of the extraction buffer, all spiked with α-AMA at varying concentrations ranging from 20 to 0.02 µg/L including a non-spiked blank. The antigen and antibody concentrations used for the cELISA were the determined optimized pair of antigen and antibody defined by the previous experiments.

## Figures and Tables

**Table 1:** Summary of the four linkage chemistries used to create hapten-protein conjugates. Atoms shown in blue are those added by the linking arm, atoms shown in black are from the toxin, and atoms shown in green are from the protein. (AMA = amanitin, CC = cyanuric chloride, MA = mixed anhydride, LB = Levena Biopharma, PERI = periodate oxidation).

Starting Material	Name	Linkage Chemistry	
β-AMA	CC	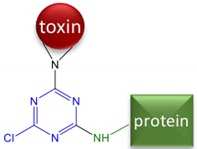 Or	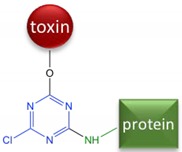
β-AMA	MA	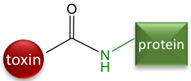	
α-AMA	LB	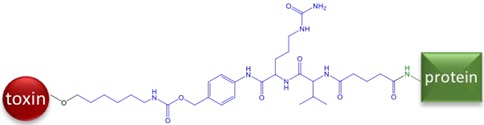	
α-AMA	PERI	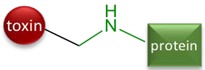	

**Table 2:** Antibody activity of rabbit sera screened against hapten-bovine serum albumin (BSA) antigens generated using four different linkage chemistries. (**a**) Relative antibody titers determined by indirect enzyme-linked immunosorbent assay (ELISA); (**b**) Preliminary IC_50_ values (µg/L) determined by screening antibody-antigen pairs by competition enzyme-linked immunosorbent assay (cELISA).

(a)	Linkage Chemistry for Coating Antigen (AMA-BSA)				(b)	Linkage Chemistry for Coating Antigen (AMA-BSA)			
Linkage Chemistry for Immunogen (AMA-KLH)	CC	MA	PERI	LB	Linkage Chemistry for Immunogen (AMA-KLH)	CC	MA	PERI	LB
63 ^1^ CC	+++^	−	−	−	63 CC	nd ^	~	~	~
64 CC	+++^	−	−	−	64 CC	nd ^	~	~	~
65 MA	+	+^	−	−	65 MA	nd	nd ^	~	~
66 MA	−	+^	−	−	66 MA	~	nd ^	~	~
53 PERI	+	+	+^	+	53 PERI	0.06 *	0.28	0.19 ^	2.98
54 PERI	+	+	+^	+	54 PERI	0.1	0.12	0.13 ^	0.42
57 LB	+	+	++	+++^	57 LB	>100	8.65	0.47	nd ^
58 LB	+	+	++	+++^	58 LB	0.48	5.97	0.07 *	nd ^

^1^ The number proceeding the description is an arbitrary identification number for each animal. nd: no α-amanitin detected; +: low activity; ++: intermediate activity; +++: high activity; −: no signal detected; ~: not tested; ^: homologous chemistries used to conjugate both the immunogen and coating antigen; *: most sensitive assay combinations.
